# Autodissemination of the entomopathogenic fungus *Metarhizium anisopliae *amongst adults of the malaria vector *Anopheles gambiae s.s.*

**DOI:** 10.1186/1475-2875-3-45

**Published:** 2004-11-28

**Authors:** Ernst-Jan Scholte, Bart GJ Knols, Willem Takken

**Affiliations:** 1 Laboratory of Entomology, Wageningen University Research Centre, Binnenhaven 7, P.O. Box 8031, Wageningen, the Netherlands; 2 Entomology Unit, Agency's laboratories Seibersdorf, International Atomic Energy Agency, A-2444 Seibersdorf, Austria

## Abstract

**Background:**

The entomopathogenic fungus *Metarhizium anisopliae *is being considered as a biocontrol agent for adult African malaria vectors. In the laboratory, work was carried out to assess whether horizontal transmission of the pathogen can take place during copulation, as this would enhance the impact of the fungus on target populations when compared with insecticides.

**Methods:**

Virgin female *Anopheles gambiae sensu stricto *were exposed to conidia whilst resting on fungus-impregnated paper. These females were then placed together for one hour with uncontaminated males in proportions of either 1:1 or 1:10 shortly before the onset of mating activity.

**Results:**

Males that had acquired fungal infection after mating indicate that passive transfer of the pathogen from infected females does occur, with mean male infection rates between 10.7 ± 3.2% and 33.3 ± 3.8%. The infections caused by horizontal transmission did not result in overall differences in survival between males from test and control groups, but in one of the three experiments the infected males had significantly shorter life spans than uninfected males (*P *< 0.05).

**Conclusion:**

This study shows that autodissemination of fungal inoculum between *An. gambiae s.s*. mosquitoes during mating activity is possible under laboratory conditions. Field studies are required next, to assess the extent to which this phenomenon may augment the primary contamination pathway (i.e. direct contact with fungus-impregnated targets) of vector populations in the field.

## Introduction

Control of the main African malaria vector *Anopheles gambiae *(Diptera: Culicidae) continues to rely heavily on application of residual insecticides, either for indoor residual house spraying [1] or bednet impregnation [2]. These approaches have been highly effective in reducing malaria morbidity and mortality [2], but associated problems regarding environmental pollution [3,4], acceptability and cost [5,6] and the now widespread and continuing development of resistance [7-10] underscore the need for alternative strategies, such as vector control with biological agents [1,11,12].

Entomopathogenic fungi are among the biological control agents used against insect pests. Interest in using the hyphomycete *Metarhizium anisopliae *against adult African malaria vectors has recently increased [13]. The fungus has proven to be highly virulent for this vector, both in the laboratory [14] as well as in the field (Scholte *et al*., in preparation). The principal method of contamination of the target insect population with the fungus is through application of conidia on indoor resting targets. However, in order to achieve the highest possible impact on the target population, it is desirable that contamination pathways other than the primary mode of contamination are utilised, for instance horizontal transmission. Horizontal transmission of pathogens within the same host/target species is called autodissemination, and this phenomenon has been suggested for biocontrol of several insect pests [15,16]. Successful transmission of *M. anisopliae *by honeybees for infection of the pollen beetle *Meligethes aeneus *[17], of *Beauveria bassiana *between adult flies of *Delia radicum *[18] and of *M. anisopliae *and *B. bassiana *between adult tsetse flies, *Glossina morsitans morsitans *[19] confirms the capability of insects to transmit fungi horizontally. Autodissemination of insecticidal biocontrol agents, such as insect-pathogenic fungi, provides an additional advantage over pesticides, as the impact on pest populations increases beyond direct contact. In several cases, autodissemination of entomopathogenic fungi within populations of insect pests, using attractant traps as the initial source of infection, has succeeded [18,20-22]. The strategy envisaged for the use of *M. anisopliae *against adult *An. gambiae *is that host-seeking females, and occasionally also males that rest indoors, will receive primary infections while resting indoors on fungus-impregnated resting targets. Under optimal circumstances, prior to death, this infection may be transmitted to conspecifics upon contact (e.g. during mating). These mosquitoes are, therefore, not infected through direct contact with fungus-impregnated materials, but indirectly, upon physical contact with infected counterparts. It is estimated that approximately half of newly hatched, virgin females take a blood meal before mating [23,24]. A female, with contaminated legs and mouthparts following the blood-feeding visit to a house containing fungus-impregnated resting targets, may contaminate male counterparts when she mates the following dusk period, thereby spreading the fungus through the population.

The objective of this study was to investigate whether adult *An. gambiae *infected with *M. anisopliae *can transmit the fungus to uncontaminated mosquitoes of the opposite sex through physical contact during the mating process.

## Materials and Methods

### Bioassays

Three bioassays were conducted to assess whether autodissemination of *M. anisopliae *can occur during the process of mating:

1) 30 fungus-contaminated virgin females and 30 uncontaminated males were placed in a standard (30 cm^3^) netting cage for one hour during the normal mating period;

2) a single fungus-contaminated virgin female and a single uncontaminated male where placed together in a glass tube for one hour during the normal mating period;

3) a single fungus-contaminated virgin female and 10 uncontaminated males were placed in a standard (30 cm^3^) netting cage for one hour during the normal mating period.

A fourth bioassay assessed whether or not:

4) males that became infected with the fungus had acquired the infective propagules from contaminated females or rather from contact with the substrate where those females had rested previously.

### Mosquitoes

The *Anopheles gambiae sensu stricto *strain used originates from Suakoko, Liberia (courtesy Prof. M. Coluzzi) and has been maintained in the Wageningen laboratories since 1989, under standardized conditions described by Mukabana *et al*. [25]. Experimental mosquitoes were four (females) or seven day old (males). Virginity of the females was assured by collecting them within 24 hrs after emergence from cages and keeping them separate from the males prior to the experiments. In all experiments, mosquito mortality was checked daily, the mosquito cadavers placed on moist filter paper and placed in Petri dishes that were sealed with parafilm. These were kept in an incubator at 27 ± 2°C and checked for fungal sporulation after three days.

### Fungus

*Metarhizium anisopliae *var. *anisopliae *isolate ICIPE30 (courtesy Dr. N.K. Maniania) was originally isolated in 1989 from a stemborer, *Busseola fusca *Fuller, near Kendu Bay, Western Kenya. Conidia were inoculated on oatmeal agar (OA) and placed in an incubator to grow. Fungal virulence was maintained by passing it through *An. gambiae *mosquitoes every five cycles after growing on OA. Third instar larvae were infected by applying dry conidia on the water surface. After 48–72 hours, moribund larvae were removed and their thorax opened to remove tissue with blastospores. These were plated on OA and placed in a dark incubator at 27°C. One week after the onset of sporulation of the colonies, conidia were harvested using a 0.05% Triton-X solution and a glass rod. The solvent with conidia was concentrated by removing the supernatant after centrifuging for three minutes at 5000 rpm. Dilutions were made using 0.05% Tween80 to obtain a conidial concentration of 10^8 ^conidia ml^-1^. Vegetable (sunflower) oil was added to obtain an 8% adjuvant oil formulation. Five ml of this suspension was pipetted evenly over a 240 cm^2 ^piece of filter paper resulting in conidial densities of 1.6 × 10^10 ^conidia m^-2^. The impregnated paper was left to dry at 20°C and 75 ± 5% RH for 48 hours and was then placed on the inside of a plastic cylinder (height 11.3 cm, diameter 3.4 cm) in such manner that the paper neatly lined the upright wall of the tube. The top of the tube was covered with netting material. This setup was used only to infect female mosquitoes. Before any contamination, the viability of the impregnated conidia was checked by placing a 1 cm^2 ^piece of the impregnated paper on a Sabourad Dextrose Agar in an incubator at 27°C in the dark for 16–20 hours. After incubation, the piece of paper was carefully removed and placed under a microscope (X 400) to determine the proportion germinated. For direct contamination of the female mosquitoes with *M. anisopliae*, around 30 individuals were placed in the cylinder for 24 hours.

### Experimental procedures

#### Bioassay 1

Thirty uninfected males were placed in a 30 cm^3 ^netting cage three hours before the onset of mating. Half an hour before (artificial) dusk, which for *An. gambiae *is the time when mating activity occurs [26], thirty contaminated females were added to this cage by releasing them from the cylinder (see above) where they had spent the previous 24 hours. By that time a large percentage of the males had the fibrillae on their antennae erect, which is considered a sign for impending mating activity [27]. One hour after introduction of the females, all males were gently removed from the cage using a 2 cm diameter glass vial and placed in a clean cage where they had access to 6% glucose *ad libitum*. The experiment was replicated three times, on different days. Control groups were treated similarly, with the difference that the paper lining the contamination tube was void of conidia. Mortality of males and females was checked daily to measure longevity. Dead mosquitoes (both sexes) were removed from the cages and placed on moist filter paper in a Petri dish, which was sealed and examined for fungal growth three days later. An additional similar experiment with only five females and five males was performed to determine whether conidia could be seen on the cuticle of the test males directly after the females were removed. Males were killed by a droplet of chloroform and placed under a microscope (X 400) and examined for attached conidia.

#### Bioassay 2

A single, uncontaminated 7-day old male was placed in a clean glass vial (diameter 2 cm, height 10 cm, sealed off with netting), to which one *M. anisopliae *contaminated female was added 30 minutes before the onset of mating activity. After one hour the couple was separated by gently removing the male, which was placed and kept in a separate glass vial until it died. A wad of cotton wool moist with 6% glucose was placed on top of the vial. Females remained in the vial and were provided with glucose in the same way. Mortality of both sexes was recorded daily. The control group consisted of an equal number of pairs that were handled equally, with the difference that the females were not infected with the fungus. This was repeated with 35 male-female pairs on three different days.

#### Bioassay 3

Ten uncontaminated 7-day old males were placed in a 30 cm^3 ^netting cage. Half an hour before the onset of mating activity, a single infected female mosquito was added to this cage with the males. After one hour each male was gently removed using a clean 2 cm diameter glass vial and kept alive individually as in bioassay 2. This was done 14 times for the test group and six times for the control group.

#### Bioassay 4

To assess whether the males in the above bioassays had acquired fungal infection from the contaminated females during the process of mating, or from resting on the substrate where fungus-contaminated females had rested previously (glass and netting), two experiments were carried out. In one experiment a total of 46 contaminated females (in two separate trials) were gently transferred to a 500 ml glass beaker, sealed of with a glass Petri-dish. The females were killed after one hour by applying a droplet of chloroform in the beaker. They were then removed and placed into a Petri-dish on a piece of moist filter paper. The dish was sealed with parafilm, to be checked three days later for fungal infection. Directly after removal of the contaminated females, a total of 47 uncontaminated males (in two separate trials) were placed in the beaker for three days, after which they were killed, removed and checked for fungal infection as described above. During the three days inside the beaker they had continuous access to a cotton wool wad moist with a 6% glucose solution. The second experiment differed only in that a standard netting cage was used instead of a glass beaker, with 64 fungus-contaminated females and 67 uncontaminated males in two separate trials.

### Data analysis

Mortality data were subjected to Kaplan-Meier pair-wise comparison survival analysis. Mosquito mortality data closely fitted the Gompertz distribution [28]. Comparisons of the average ages at the time of death were calculated and analyzed using GLM analysis. All analyses were carried out by using Genstat 7.0.

## Results

In all four bioassays, female mosquitoes that had been exposed to conidia in the cylinder setup died significantly faster than the control females (F = 104.4; p < 0.001), with an average of 96.4 ± 2.0% of the cadavers having sporulating *M. anisopliae*.

**Figure 1:**
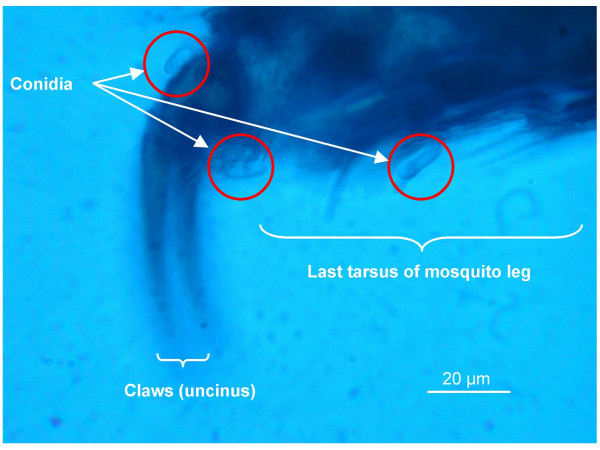
Conidia of the entomopathogenic fungus *Metarhizium anisopliae *on the lower part of a male *Anopheles gambiae s.s*. leg, after having been horizontally transferred from an infected female during copulation.

All three replicates of bioassay 1 indicated transfer of the fungus from contaminated females to uninfected males, with an average male infection rate of 26.1 ± 5.3% (Table 1). There was no difference in survival between the males of the control and test groups (F = 0.30; p = 0.5844), but when the group of test males was split into those that had been infected and those that had not, survival analysis showed a difference in survival approaching significance (F = 2.73; p = 0.098). Under a compound microscope, conidia were observed on four of the five males. Most were found on the lower parts (tibia, tarsi, uncinus (claws) and arolium) of the first and second pair of legs (Figure 1). A few conidia were found attached to the hairs of the tip of the wings. No conidia were found on the head, thorax, abdomen or the hind legs. Per mosquito 0–25 conidia were found. Female mosquitoes that had spent 24 hrs on fungus-impregnated paper had conidia on legs, tips of their wings and mouthparts, but not on the thorax or abdomen.

**Table 1:** Autodissemation of *M. anisopliae *from females to males *An. gambiae s.s*.. The proportions of male mosquitoes infected are shown with the differences in survival of fungus-infected males compared to uninfected males within the test groups.

Bioassay	Ratio	Average %(± SEM^a^) of males infected	Survival analysis (Kaplan Meier)	Average age of males at time of death (days)	
				
				infected	uninfected
1	1:1	26.1 ± 5.3	F = 2.73; p = 0.098	10.3 ± 0.7	18.6 ± 0.8^b^
2	1:1	34.0 ± 0.6	F = 3.39; p = 0.065	11.3 ± 1.9	15.6 ± 1.6^b^
3	1:10	10.7 ± 3.2	F = 13.02; p = 0.001	13.1 ± 1.3	17.9 ± 0.6^b^

^a^: SEM = standard error of mean

^b^: Significant difference (p < 0.05) between infected and uninfected males using GLM analysis (Genstat 7.0).

In the second bioassay, with individual pairs in glass vials, an average of 34.2 ± 0.6% of the males acquired and died of the fungal infection. Survival of these males as a group was not significantly different from the control group (F = 1.27; p = 0.259). When the males from the test group were split into those that became infected and those that did not, a decrease in survival of the infected test males compared to the uninfected males was observed, although this effect was not significant (F = 3.39; p = 0.065). However, the average age at death of 11.3 ± 1.9 days of the fungus-infected males was significantly lower than the 15.6 ± 1.6 days of the uninfected test males (p = 0.001).

In the third bioassay, horizontal transmission occurred in nine of the 14 replicates (64.3%). In those nine trials an average of 16.7 ± 3.7% of the 10 males acquired an infection. Calculated over the 14 trials, the infection rate was 10.7 ± 3.2%. As in the other two bioassays, there was no significant difference in survival rates between the test and control groups (F = 2.19; p = 0.139). However, those males in the test group that were found with sporulating fungus died significantly faster than uninfected males (F = 13.02; p = 0.001) with the average age at the time of death of 8.4 ± 1.1 days for infected and 17.9 ± 0.6 days for uninfected males, respectively.

None of the 114 males in bioassay 4 were found infected with the fungus.

## Discussion

It is generally believed that fungal dissemination within a host population occurs due to activities and movements of the host. The fungus can exploit host behaviour like trophallaxis, tactile communication, grooming (in social insects) [29,30] and mating [31] to spread through a host population. Taking into account the physiological state of the females and the natural display of behaviour at the time of the bioassays, it is assumed that the observed autodissemination of *M. anisopliae *from female to male *An. gambiae s.s*. was the result of mating. This is strongly supported by the findings from experiment 4 where none of the males that had stayed on the surface area where fungus-contaminated females had rested previously acquired an infection. The average age at death of fungus-infected mosquitoes was quite high when compared to mosquito survival in Scholte *et al*. [14]. This is probably due to the relatively low level (a maximum of 25 conidia) of inoculum transferred. From those mosquitoes that were checked under the microscope for the presence of conidia, four out of five males contained conidia. It is thus likely that many males become contaminated, but that only a relatively low proportion of these males will actually succumb to the infection: in many cases the number of conidia was low, resulting in marginal infections that were successfully countered by the immune responses staged by the males.

In order to achieve the highest possible impact of the fungus on the mosquito population, it is desirable that other pathways besides the primary mode of (direct) contamination are utilized. The results of this study show that under laboratory conditions horizontal transmission can occur, which suggests that it may occur in the field. When these experiments were carried out, it was presumed that predominantly females would be infected directly from the indoor resting targets in the field. From a recent field experiment (Scholte *et al*., in preparation), however, it was found that a large proportion (44.9%) of the *An. gambiae s.l*. found indoors were males. This suggests that not only females can deliver fungal inoculum to uninfected males, but that also infected males may infect uninfected females. Further research is needed to determine to what extent this secondary pathway of fungal contamination may contribute to spreading the fungus within mosquito field populations.

## Conclusion

This study has shown that horizontal transfer of fungal inoculum between mosquitoes is possible during copulation and may contribute to spreading of the fungus within target mosquito populations in the field. However, since conditions under which horizontal transmission is likely to occur are quite specific, field experimentation is required to measure the real impact that autodissemination may have. For now it is concluded that the relatively low infection levels recorded in this study suggest that the impact of biological control with *M. anisopliae *against African anophelines will predominantly depend on direct contamination of adult mosquitoes from conidia-impregnated resting targets such as walls, ceilings and sheets.

## Authors' contribution

E-JS was directly involved in the experimental work, analysis of the data and drafting of the manuscript. BGJK conceived of the study, obtained funding for it in collaboration with WT and revised the manuscript prior to submission.
